# BactQuant: An enhanced broad-coverage bacterial quantitative real-time PCR assay

**DOI:** 10.1186/1471-2180-12-56

**Published:** 2012-04-17

**Authors:** Cindy M Liu, Maliha Aziz, Sergey Kachur, Po-Ren Hsueh, Yu-Tsung Huang, Paul Keim, Lance B Price

**Affiliations:** 1 Division of Pathogen Genomics, Translational Genomics Research Institute, 3051 W. Shamrell Blvd., Suite 106, Flagstaff, AZ 86001, USA; 2 Center for Microbial Genetics and Genomics, Applied Research & Development Building,, Northern Arizona University, 1298 S. Knoles Drive, Flagstaff, AZ, 86011, USA; 3 Departments of Laboratory Medicine and Internal Medicine, National Taiwan University Hospital, National Taiwan University College of Medicine, No. 7, Chung-Shan South Road, Taipei, Taiwan; 4 Department of Internal Medicine, Far Eastern Memorial Hospital, No.21, Nanya S. Rd., New Taipei City, Taiwan; 5 Current Address: Ross University School of Medicine, 630 US Highway 1, North Brunswick, NJ, 08902, USA

## Abstract

**Background:**

Bacterial load quantification is a critical component of bacterial community analysis, but a culture-independent method capable of detecting and quantifying diverse bacteria is needed. Based on our analysis of a diverse collection of 16 S rRNA gene sequences, we designed a broad-coverage quantitative real-time PCR (qPCR) assay—BactQuant—for quantifying 16 S rRNA gene copy number and estimating bacterial load. We further utilized *in silico* evaluation to complement laboratory-based qPCR characterization to validate BactQuant.

**Methods:**

The aligned core set of 4,938 16 S rRNA gene sequences in the Greengenes database were analyzed for assay design. Cloned plasmid standards were generated and quantified using a qPCR-based approach. Coverage analysis was performed computationally using >670,000 sequences and further evaluated following the Minimum Information for Publication of Quantitative Real-Time PCR Experiments (MIQE) guidelines.

**Results:**

A bacterial TaqMan® qPCR assay targeting a 466 bp region in V3-V4 was designed. Coverage analysis showed that 91% of the phyla, 96% of the genera, and >80% of the 89,537 species analyzed contained at least one perfect sequence match to the BactQuant assay. Of the 106 bacterial species evaluated, amplification efficiencies ranged from 81 to 120%, with *r*^*2*^-value of >0.99, including species with sequence mismatches. Inter- and intra-run coefficient of variance was <3% and <16% for Ct and copy number, respectively.

**Conclusions:**

The BactQuant assay offers significantly broader coverage than a previously reported universal bacterial quantification assay BactQuant in vitro performance was better than the *in silico* predictions.

## Background

Scientists today are studying bacterial communities from diverse habitats, hosts, and health conditions based on the 16 S rRNA gene [1,2]. To date, most studies have focused on qualitative characterization based on the relative abundances of community bacterial groups [3-5]; however, quantitative characterization—i.e., measurement of the total bacterial load—provides valuable and complementary information when combined with these qualitative data [6]. Traditional culture-based approaches for quantifying bacterial load are inherently limited for assessing the complex bacterial communities that exist in many clinical and environmental samples. Likewise, standard culture-based methods are ineffective for quantifying many fastidious and uncultivable bacterial species [7].

Among culture-independent approaches, quantitative real-time PCR (qPCR) is currently best suited for measuring bacterial load, because of its intrinsic quantitative capability, ease of use, and flexibility in assay design [8,9]. Using the qPCR platform, we can design an assay capable of concurrently detecting and quantifying all unique bacteria that constitutes a complex community. Furthermore, by utilizing 16 S rRNA gene as the target of a broad coverage qPCR assay, results from the qPCR evaluation can be easily combined with 16 S rRNA gene-based qualitative characterization to fully describe the community of interest.

In the current paper, we present our design and validation of a broad-coverage quantitative real-time PCR assay—BactQuant—for quantifying 16 S rRNA gene copy number and estimating bacterial load. To accomplish this, we have employed a novel nucleotide distribution-based approach to effectively summarize a large 16 S rRNA gene sequence dataset for qPCR assay design. We further addressed a general limitation of the qPCR platform—the normalization of in-run quantitative standards using fluorimetric or spectrometric methods—by developing an alternative qPCR-based method for quantifying plasmid standards. Lastly, we have complemented standard qPCR assay validation following MIQE guideline [10] with extensive *in silico* analysis using >670,000 16 S rRNA gene sequences from the Ribosomal Database Project [11].

## Methods

### Design of 16 S rRNA gene quantitative real-time PCR assay

Pre-aligned 16 S rRNA gene sequences (n = 4,938) were downloaded from the core set of the Greengenes database [12]. The alignment was analyzed to generate an output of nucleotide distribution—i.e., the summary of allele frequency at each nucleotide position in the 16 S rRNA gene multiple sequence alignment file—and diversity score using a 3% gap-filter setting and the Simpson’s Diversity Index, respectively.

#### Assay Design

The nucleotide distribution was examined to identify a conserved 500 bp region for assay design. In designing the assays, we applied the following rules: 1) primer sequences cannot have more than three degenerate bases and 2) the probe sequence cannot have any degenerate bases. The primer T_m_ was calculated using salt adjusted calculation from the online tool OligoCalc [13] and the probe T_m_ was calculated using the Primer Probe Test Tool for TaqMan® MGB quantification from the Primer Express® Software for Real-Time PCR version 3.0 (Applied Biosystems, Carlsbad, CA, USA) (Table1).

**Table 1:** Primer and probe sequences of BactQuant, the new 16 S rRNA gene-based quantitative real-time PCR (bold letters denotes degenerate base)

**BactQuant**		**Tm *(°C)***	***E. coli* region**
Forward Primer	5′- CCTACGGG**D**GGC **W**GCA-3′	55.9–58.4	*341–356*
Reverse Primer	5′- GGACTAC**HV**GGGT **M**TCTAATC -3′	57.5–63.3	*786–806*
Probe	(6FAM) 5′-CAGCAGCCGCGGTA-3′ (MGBNFQ)	68.0	*519–532*

### Computational analysis of assay specificity and coverage

 A. Specificity analysis. Specificity check was performed in GenBank using megablast against human, mouse, and fungal sequences from the nucleotide collection (nr/nt) [14].

 B. Collection and identification of bacterial 16 S rRNA gene sequence eligible for *in silico* coverage analysis. All 16 S rRNA gene sequence data used in the *in silico* coverage analysis were downloaded from the Ribosomal Database Project (RDP) Release 10 Update 20 [11]. Briefly, all bacterial 16 S rRNA gene sequences that were of “Good” quality and had a length of 1200 bp or greater were extracted from RDP, along with taxonomic metadata and sequence IDs. Additionally, the *Escherichia coli* position data was kindly provided by staff at the RDP. The downloaded sequences were filtered based on *E. coli* position. Only sequences with data present in the qPCR assay amplicon of interest were considered to be eligible for sequence matching for the particular qPCR assay. Numerical and taxonomic coverage analysis was performed for the BactQuant assay and a published qPCR assay [15] by developing a web service for the RDP Probe Match Tool for sequence matching.

 C. Overview of sequence matching analysis for determining assay coverage. All sequence matching for the *in silico* coverage analysis was performed using two conditions: a) perfect match of full-length primer and probe sequences and b) perfect match of full-length probe sequence and the last 8 nucleotides of primer sequences at the 3´ end. For each sequence matching condition, the *in silico* coverage analysis was performed at three taxonomic levels: phylum, genus, and species, as well as for all sequences eligible for sequence matching. The remaining taxonomic levels were omitted due to the large amounts of missing and inconsistent data. Details of *in silico* coverage analyses are as follows:

 D. Numerical coverage analysis. At each analysis level, unique operational taxonomic unit (OTU), i.e., each unique taxonomic group ranging from unique phyla to unique species, containing at least one sequence that is a sequence match (i.e., “match”) for all three components of the assay of interest were identified using the following requirement: [Forward Primer Perfect Match](union)[Reverse Primer Perfect Match](union)[Probe Perfect Match]. The *in silico* coverage analysis was performed in a stepwise fashion, beginning with all eligible sequences, then proceeding to analysis at the species-, genus-, and phylum-level. At each step, the taxonomic identification of each sequence was generated by concatenation of relevant taxonomic data (e.g., for species-level analysis, a unique taxonomic identification consisting of concatenated Phylum-Genus- *species* name was considered as one unique species). The sequence IDs were used in lieu of a taxonomic identification for the first analysis step, which included all eligible sequences. The stepwise numerical coverage analysis was performed as follows: all eligible sequences underwent sequence matching with all three components of the assays of interest using a select matching condition (i.e., the stringent or the relaxed criterion). The sequence IDs of matched sequences were assigned and binned as Assay Perfect Match sequence IDs. For this first analysis step, the numerical coverage was calculated using the total number of sequences with Assay Perfect Match sequence IDs as the numerator and the total number of eligible sequences as the denominator. Next, at the species-level, all sequences assigned as Assay Perfect Match sequence IDs were dereplicated based on the concatenated Phylum-Genus- *species* taxonomic identifications. Species-level numerical coverage was then calculated using the total number of dereplicated taxonomic identifications as the numerator. Denominator was calculated using the dereplicated Phylum-Genus- *species* taxonomic identifications from all eligible sequences. As a result of the logic of this analysis pipeline, a species (i.e., a group of sequences sharing the same unique Phylum-Genus- *species* designation) was considered an assay sequence match and thus “covered”, when at least one Assay Perfect Match sequence ID was in the species group. The numerical coverage analysis was repeated on the genus-level using the dereplicated Phylum-Genus taxonomic identifications from the Assay Perfect Match sequence IDs bin (numerator) and from all eligible sequences (denominator), and lastly, on the phylum-level using Phylum taxonomic identifications. To facilitate calculation of assay coverage, two ambiguous phyla, “Bacteria Insertia Sedis” and “Unclassified Bacteria” were excluded from the phylum-level analysis. Sequences with genus, species, and strain names containing “unclassified” were included in the numerical coverage analyses due to their high abundance.

 E. Taxonomic coverage analysis. The *in silico* taxonomic coverage analysis was performed to generate a detailed output consisting of the taxonomic identifications that were covered or “uncovered” (i.e., no sequence match) at multiple taxonomic levels. A step-wise approach was again utilized for this analysis, beginning with all eligible sequences, performed as follows: First, the Assay Perfect Match sequence IDs were subtracted from the sequence IDs from all eligible sequences, with the resultant sequences assigned and binned as Assay Non-Perfect Match sequence IDs. Next, on the species-level, the Phylum-Genus- *species* taxonomic identifications of all eligible sequences was first dereplicated, from which the “covered” species taxonomic identifications were subtracted. Species-level taxonomic coverage was then presented as a list of concatenated taxonomic identification of the covered and uncovered species. This was repeated with the genus- and phylum-level taxonomic identifications for genus- and phylum-level taxonomic coverage analyses. Output of taxonomic identifications from analysis using all eligible sequences was not presented in this manuscript due to its extensive size but is available in Additional file 1: Figure S 1.

**Figure 1:**
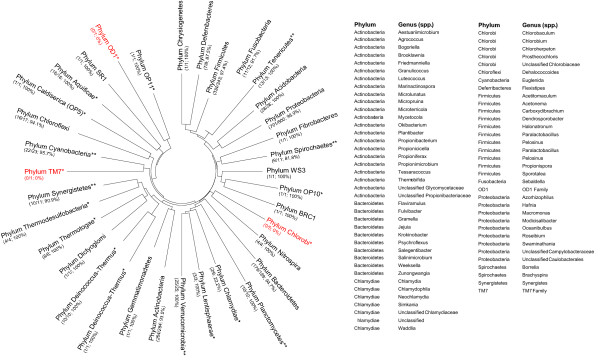
**Results from*in silico*coverage analysis of the BactQuant assay using the stringent criterion against 1,849 genera and 34 phyla showing broad coverage.** The number of covered genus for each phylum analyzed ( *left*) and the list of all uncovered genera ( *right*) are shown. On the circular 16 S rRNA gene-based maximum parsimony phylogeny ( *left*), each of the covered ( *in black*) and uncovered ( *in red*) phylum by the BactQuant assay is annotated with the genus-level numerical coverage in parenthesis below the phylum name. Each genus-level numerical coverage annotation consists of a numerator (i.e., the number of covered genus for the phylum), a denominator (i.e., the total number of genera eligible for sequence matching for the phylum), and a percentage calculated using the numerator and denominator values. Comparison with the published assay is presented for each phylum as notations of a single asterisk (*) for phylum not covered by the published assay and as a double asterisk (**) for phylum with <50% of its genera covered by the published qPCR assay. The phylum and genus taxonomic identifications of all genera not covered by the BactQuant assay are also presented ( *right*) (Unc = Unclassified).

 F. Assay comparison using results from the *in silico* analyses. Results from the *in silico* analyses were summarized for assay comparison as follows: The numerical coverage for the BactQuantand published qPCR assays were calculated at three taxonomic levels, as well as for all eligible sequences using both sequence matching conditions and presented as both the numerator and denominator, and percent covered calculated as the numerator divided by the denominator. This was presented in Table2. Additional comparison of the taxonomic coverage was performed by superimposing the genus-level numerical coverage of the BactQuant assay for each phylum onto a maximum parsimony phylogenetic tree. Construction of the phylum-level phylogenetic tree was performed using MEGA4 with representative full-length 16 S rRNA gene sequences from each of the 34 phyla analyzed [16]. In addition, each phylum was annotated as not covered or poorly covered by the published qPCR assay if the phylum was uncovered or if >50% of the genera within the phylum were uncovered, respectively. A list of the uncovered genera by phylum for the BactQuant assay was also generated. Comparison results using the stringent and relaxed criterion were presented in Figure1 and Additional file 2: Figure S1, respectively.

**Table 2:** Results from numerical coverage analysis performed by comparing primer and probe sequences from BactQuant and the published qPCR assays against >670,000 16 S rRNA gene sequences from RDP

	**BactQuant**	**Published qPCR Assay**	**Coverage Improvement**
A. Perfect match using full length primers and probe			
Phyla	**91.2%** (31/34)	**61.8%** (21/34)	+ 29.4%
Genus	**96.2%** (1778/1849)	**80.3%** (1485/1849)	+15.8%
Species*	**83.5%** (74725/89537)	**66.3%** (59459/89646)	+17.2%
All Sequences*	**78.0%** (524118/671595)	**60.9%** (409584/672060)	+17.1%
B. Perfect match using 8-nt primers with full length probe			
Phyla	**91.2%** (31/34)	**67.7%** (23/34)	+23.5%
Genus	**97.7%** (1806/1849)	**82.1%** (1518/1849)	+15.6%
Species*	**89.1%** (79759/89537)	**70.9%** (63533/89646)	+18.2%
All Sequences*	**84.4%** (566685/671595)	**65.6%** (441017/672060)	+18.8%

The *in silico* analysis was performed using two sequence matching conditions.

*The difference in number of sequences eligible for *in silico* evaluation is due to the difference in primer lengths and locations of the two assays.

### Quantification and normalization of cloned plasmid standards

#### Overview

To obtain accurately quantified plasmid standards for validation the BactQuant assay, a 10^9^ copies/μl plasmid stock was quantified using a qPCR assay targeting portion of the vector using the second derivative maximum analysis algorithm on the LightCyler platform. The resultant crossing point value (i.e., Cp-value) is used in plasmid normalization. The details are as follows:

#### Generation of normalized 16 S rRNA gene plasmid standards

Amplification of the full 16 S rRNA gene was performed using *E. coli* genomic DNA as the template and 16 S rRNA gene primers 27 F and 1492R as previously described [17]. Visualization of PCR amplicon was performed using gel electrophoresis with SYBR 2% agarose gel. The resultant PCR amplicons were immediately used as the target gene insert with the TOPO® TA Cloning® Kit (with pCR®2.1 TOPO® vector) (Invitrogen Corp., Carlsbad, CA, USA) following the manufacturer’s instructions. The resultant propagated cloned plasmids were purified using the QIAprep Spin Miniprep Kit (Qiagen Inc., Valencia, CA, USA). Sequence verification of the purified plasmids containing the 16 S rRNA gene insert was performed with capillary electrophoresis using BigDye® Terminator v3.1 Cycle Sequencing Kit on the 3130 Genetic Analyzer platform (Applied Biosystems, Carlsbad, CA, USA). Quantification of the cloned plasmids was performed by analyzing three 10-fold dilutions using the vector qPCR assay. Normalization was performed using the dilution factor 2^ΔCp^, where ΔCp = 10 – (Cp value of non-normalized cloned plasmids).

### Pan-bacterial qPCR assay optimization and initial specificity check

#### Assay optimization

Using the normalized plasmid standards, different primer and probe titrations were tested on the on the 7900HT Real Time PCR System (Applied Biosystems) and evaluated based on reaction efficiency and assay dynamic range for 10 μl and 5 μl reaction volumes. For 10 μl and 5 μl reactions, the optimized conditions included 1 μl of template into 9 μl and 4 μl of reaction mix, respectively, with the final reaction containing 1.8 μM of each forward and reverse primer, 225 nM the TaqMan® probe, 1X Platinum® Quantitative PCR SuperMix-UDG w⁄;ROX (Invitrogen Corp.) and molecular-grade water. Irrespective of reaction volume, each experiment included an in-run standard curve (10^2^–10^8^ in 10-fold serial dilutions) and no-template controls performed in triplicate. Amplification and real-time fluorescence detections were performed on the 7900HT Real Time PCR System (Applied Biosystems) using the following PCR conditions: 3 min at 50°C for UNG treatment, 10 min at 95°C for *Taq* activation, 15 s at 95°C for denaturation and 1 min at 60°C for annealing and extension x 40 cycles. Cycle threshold value (i.e., Ct value) for each 16 S qPCR reaction were obtained using a manual Ct threshold of 0.05 and automatic baseline in the Sequence Detection Systems v2.3 software (Applied Biosystems).

#### Initial specificity check against human and fungal genomic DNA

Using the optimized assay condition, the newly designed assay was tested against 1 ng, 100 pg, and 10 pg of human genomic DNA (Promega, Madison, WI, USA), *C. albicans* genomic DNA (American Type Culture Collection, Manassas, VA, USA), the normalized plasmid standards in triplicate reactions.

### Laboratory analysis of assay performance using diverse bacterial genomic DNA

To assess our assay performance against diverse bacteria, we tested our assay against a diverse collection of bacterial genomic DNA to determine the assay efficiency and correlation coefficients. The details are as follows:

#### Bacterial strains

*Arsenophonus nasoniae* ATCC 49151 *, Budvicia aquatica* ATCC 51341, *Buttiauxella gaviniae* ATCC 51604, *Cedecea davisae* ATCC 33431 *, Cellvibrio gilvus* ATCC13127, *Citrobacter freundii* ATCC 8090, *Clostridium difficile* ATCC 9689, *Cronobacter aerogenes* ATCC 13048, *Ewingella americana* ATCC 33852 *, Edwardsiella tarda* ATCC 15947, *Escherichia vulneris* ATCC 33821, *Hafnia alvei* ATCC 29926, *Ewingella americana* ATCC 33852 *, Klebsiella oxytoca* ATCC 49131, *Kluyvera ascorbata* ATCC 33433, *Leclericia adecarboxylata* ATCC 700325, *Leminorella richardii* ATCC 33998, *Moellerella wisconsensis* ATCC 35621, *Morganella morganii* ATCC 25830, *Obesumbacterium proteus* ATCC 12841, *Pantoea agglomerans* ATCC 27155, *Photorhabdus asymbiotica* ATCC 43950, *Plesiomonas shigelloides* ATCC 14029, *Pragia fontium* ATCC 49100, *Proteus mirabilis* ATCC 29906 *, Providencia rustigianii* ATCC 33673, *Pseudomonas aeruginosa* ATCC 27853, *Pseudomonas andersonii* ATCC BAA-267, *Pseudomonas anguilliseptica* ATCC 33660, *Pseudomonas azotofixans* ATCC BAA-1049, *Pseudomonas fragi* ATCC 4973, *Pseudomonas lundensis* ATCC 49968, *Pseudomonas luteola* ATCC 43273, *Pseudomonas mendocina* ATCC 25411, *Pseudomonas monteilii* ATCC 700476, *Pseudomonas mosselii* ATCC BAA-99, *Pseudomonas otitidis* ATCC BAA-1130, *Pseudomonas pseudoalcaligenes* ATCC 17440, *Psuedomonas putida* ATCC 12633, *Pseudomonas stutzeri* ATCC 17588, *Pseudomonas taetrolens* ATCC 4683, *Rahnella aquatilus* ATCC 33071, *Raoultella ornithinolytica* ATCC 31898 *, Shigella dysenteriae* ATCC 13313, *Salmonella enterica* ATCC 13076, *Serratia liquefaciens* ATCC 27592, *Tatumella ptyseos* ATCC 33301, *Trabulsiella guamensis* ATCC 49492, *Yersinia enterocolitica* ATCC 9610, and *Yokenella regensburgei* ATCC 43001 were obtained from the American Type Culture Collection (Manassas, VA, USA). Bacterial propagation and enrichment were performed under the appropriate condition for each bacterial strain following ATCC recommendations.

#### Extraction of bacterial genomic DNA

Extraction using the enriched broth was performed using ZR Fungal/Bacterial DNA MiniPrepTM (Zymo Research, Irvine, CA, USA) following the manufacturer’s instruction. Elution of the purified genomic DNA was performed using 100 μl of 1X TE buffer.

#### Other sources of bacterial genomic DNA

Genomic DNA from *Bacteroides fragilis* ATCC 25285 *, Bacteorides ureolyticus* GS-15 ATCC 43606, *Borrelia burgdorferi* strain B31 ATCC 35210 *, Campylobacter jejuni* ATCC 700819 *, Chlaymydia trachomatis* ATCC VR-348B *, Chlamydophila pneumoniae* ATCC VR-1360D *, Fusobacterium nucleatum* ATCC 25586 *, Pectobacterium atrosepticum* ATCC BAA-672 *, Pseudomonas syringae* ATCC 11355 *, Streptomyces violaceoruber* ATCC BAA-471 *, Thermus thermophilus* ATCC BAA-163 *, Treponema denticola* ATCC 35405 *, and Vibrio cholera* ATCC 39315 were obtained from the American Type Culture Collection. DNA from *Mycobacterium avium, subsp. Avium, Mycobacetrium abscessus, Mycobacterium bovis, Mycobacterium chelonae, Mycobacterium gastri, Mycobacterium gordonae, Mycobacterium fortuitum, Mycobacterium kansasii, Mycobacterium marinum, Mycobacterium nonchromogenicum, Mycobacterium phlei, Mycobacterium smegmatis, Mycobacterium vaccae, and Mycobacterium xenopi* were kindly provided by National Taiwan University, Taipei, Taiwan. DNA from clinical isolates of *Acinetobacter baumannii, Klebsiella pneumoniae, Burkholderia pseudomallei, Coxiella burnetti, Enterobacter cloacae, Enterococcus faecium, Escherichia coli, Francisella tularensis, Haemophilus influenzae, Legionella pneumophila, Listeria monocytogenes, Moraxella catarrhalis, Neisseria gonorrhoeae, Pseudomonas aeruginosa, Salmonella enterica subsp. enterica serovar gallinarum*, *Staphylococcus arlettae, Staphylococcus capitis, Staphylococcus cohnii, Staphylococcus epidermidis, Staphylococcus equorum, Staphylococcus hominis, Staphylococcus haemolyticus, Staphylococcus kloosii, Staphylococcus lugdunensis, Staphylococcus saprophyticus, Staphyloccocus xylosus, Streptococcus agalactiae, Streptococcus pneumoniae, and Viridans Streptococcus* and were kindly provided by a project supported by NIH/NIAID U01AI066581 at the Translational Genomics Research Institute, Flagstaff, AZ, USA.

#### Experimental design

For sensitivity and efficiency analysis, bacterial genomic DNA from each species was analyzed in three 10-fold serial dilutions in triplicate reactions using the optimized 16 S qPCR conditions as described above.

#### Data analysis

For each species tested, reaction efficiency and correlation coefficient were calculated using the data from tests against three 10-fold serial dilutions and presented in Table3. Sequence comparison analysis was performed by aligning the assay primer and probe sequences with 16 S rRNA gene sequences of the five uncovered species: *Borrelia burgdorferi* (Genbank Accession No. X98226), *Cellvibrio gilvus* (Genbank Accession No. GU827555.1), *Escherichia vulneris* (Genbank Accession No. AF530476), *Chlamydia trachomatis* (Genbank Accession No. NR025888), and *Chlamydophila pneumoniae* (Genbank Accession No. CPU68426) in SeqMan®. Amplification profile of the five uncovered species were annotated with results from the sequence comparison and presented in Additional file 3: Figure S 3A-E.

**Table 3:** **The efficiency and*r***^***2***^**-value results from laboratory evaluation of the BactQuant assay using genomic DNA from ATCC strains and clinical isolates belonging to 106 unique bacterial species spanning eight bacterial phyla**

**Species Name**	**Reaction efficiency**	***r***^***2***^**-value**
***Streptomyces violaceoruber***	93%	*>0.999*
***Mycobacterium abscessus***	110%	*>0.999*
***Mycobacterium bovis***	106%	*>0.996*
***Mycobacterium chelonae***	101%	*>0.999*
***Mycobacterium gastri***	104%	*>0.999*
***Mycobacterium gordonae***	104%	*>0.999*
***Mycobacterium fortuitum***	93%	*>0.999*
***Mycobacterium kansasii***	107%	*>0.999*
***Mycobacterium marinum***	110%	*>0.990*
***Mycobacterium nonchromogenicum***	101%	*>0.999*
***Mycobacterium phlei***	104%	*>0.999*
***Mycobacterium smegmatis***	105%	*>0.999*
***Mycobacterium vaccae***	120%	*>0.999*
***Mycobacterium xenopi***	112%	*>0.999*
***Bacteroides ureolyticus***	92%	*>0.999*
***Bacteroides fragilis***	82%	*>0.993*
***Chlamydia trachomatis***	N/A	*N/A*
***Chlamydophila pneumoniae***	N/A	*N/A*
***Thermus thermophilus***	97%	*>0.999*
***Clostridium difficile***	88%	*>0.987*
***Listeria monocytogenes***	104%	*>0.999*
***Staphylococcus arlettae***	96%	*>0.998*
***Staphylococcus capitis***	95%	*>0.993*
***Staphylococcus cohnii***	104%	*>0.999*
***Staphylococcus epidermidis***	96%	*>0.999*
***Staphylococcus equorum***	85%	*>0.997*
***Staphylococcus hominis***	108%	*>0.999*
***Staphylococcus haemolyticus***	90–104%	*>0.999*
***Staphylococcus kloosii***	98%	*>0.999*
***Staphylococcus lugdunensis***	94%	*>0.999*
***Staphylococcus saprophyticus***	87–98%	*>0.999*
***Staphylococcus xylosus***	81–100%	*>0.999*
***Streptococcus agalactiae***	98%	*>0.998*
***Streptococcus pneumoniae***	98%	*>0.999*
***Streptococcus viridans***	103%	*>0.999*
***Enterococcus faecium***	91–111%	*>0.999*
***Enterococcus faecalis***	90–100%	*>0.998*
***Fusobacterium nucleatum***	90%	*>0.999*
***Burkholderia pseudomallei***	103%	*>0.999*
Coxiella burnetti*	100%	*>0.998*
***Francisella tularensis***	100%	*>0.999*
***Legionella pneumophila***	98%	*>0.999*
***Neisseria gonorrhoeae***	95%	*>0.997*
***Pseudomonas aeruginosa***	90–100%	*>0.999*
***Pseudomonas mendocina***	93%	*>0.999*
***Pseudomonas andersonii***	90%	*>0.999*
***Pseudomonas otitidis***	93%	*>0.999*
***Pseudomonas stutzeri***	86%	*>0.999*
***Pseudomonas monteilii***	88%	*>0.999*
***Pseudomonas azotofixans***	84%	*>0.999*
***Pseudomonas mosselii***	92%	*>0.999*
***Pseudomonas luteola***	91%	*>0.999*
***Pseudomonas putida***	90%	*>0.999*
***Pseudomonas fluorescens***	96%	*>0.999*
***Pseudomonas taetrolens***	89%	*>0.999*
***Pseudomonas fragi***	93%	*>0.999*
***Pseudomonas syringae***	95%	*>0.999*
***Pseudomonas pseudoalcaligenes***	93%	*>0.999*
***Pseudomonas lundensis***	93%	*>0.999*
***Pseudomonas anguiliseptica***	93%	*>0.999*
***Cellvibrio gilvus***	92%	*>0.999*
***Acinetobacter baumannii***	100–105%	*>0.999*
***Arsenophonus nasoniae***	87%	*>0.998*
***Budvicia aquatica***	88%	*>0.999*
***Buttiauxella gaviniae***	107%	*>0.999*
***Cedecea davisae***	97%	*>0.999*
***Citrobacter freundii***	95%	*>0.999*
***Cronobacter sakazakii***	96%	*>0.999*
***Edwardsiella tarda***	106%	*>0.999*
***Enterobacter cloacae***	89–111%	*>0.999*
***Enterobacter aerogenes***	107%	*>0.998*
***Escherichia vulneris***	93%	*>0.999*
***Escherichia coli***	91–96%	*>0.999*
***Ewingella americana***	97%	*>0.999*
***Haemophilus influenzae***	91–110%	*>0.999*
***Hafnia alvei***	93%	*>0.999*
***Klebsiella oxytoca***	93%	*>0.999*
***Klebsiella pneumoniae***	95–100%	*>0.999*
***Kluyvera ascorbata***	100%	*>0.999*
***Leclercia adecarboxylata***	93%	*>0.999*
***Leminorella richardii***	94%	*>0.999*
***Moellerella wisconsensis***	93%	*>0.999*
***Moraxella catarrhalis***	91–106%	*>0.999*
***Morganella morganii***	95%	*>0.999*
***Obesumbacterium proteus***	114%	*>0.994*
***Pantoea agglomerans***	93%	*>0.999*
***Pectobacterium atrosepticum***	90%	*>0.999*
***Photorhabdus asymbiotica***	96%	*>0.999*
***Plesiomonas shigelloides***	93%	*>0.999*
***Pragia fontium***	100%	*>0.998*
***Proteus mirabilis***	98%	*>0.999*
***Providencia rustigianii***	93%	*>0.999*
***Rahnella aquatilis***	92%	*>0.999*
***Raoultella ornithinolytica***	94%	*>0.999*
***Salmonella enterica***	101%	*>0.999*
***Salmonella enterica subsp. enterica serovar gallinarum***	95%	*>0.998*
***Serratia liquefaciens***	94%	*>0.999*
***Shigella dysenteriae***	98%	*>0.999*
***Tatumella ptyseos***	101%	*>0.999*
***Trabulsiella guamensis***	95%	*>0.999*
***Yokenella regensburgei***	96%	*>0.999*
***Yersinia enterocolitica***	98%	*>0.999*
***Campylobacter jejuni***	89%	*>0.999*
***Vibrio cholerae***	85%	*>0.996*
***Borrelia burgdorferi***	90%	*>0.999*
***Treponema denticola***	82%	*>0.999*

*No 16 S rRNA gene sequence available in the Ribosomal Database Project.

### Laboratory quantitative assay validation using pure plasmid standards and mixed templates

#### Assay quantitative validation

For the assay quantitative validation, we followed the Minimum Information for publication of Quantitative real-time PCR Experiments, or the MIQE guidelines whenever applicable [10]. The MIQE guidelines were complemented with additional tests to determine assay performance in the presence of background fungal and human genomic DNA. In our experimental design, we included seven template conditions: plasmid standards alone and plasmid standards with 0.5 ng *C. albicans* genomic DNA (ATCC) and with 0.5 ng, 1 ng, 5 ng, and 10 ng of human genomic DNA per reaction in 10 μl reactions and plasmid standards alone in 5 μl reactions. For each condition assessed, three qPCR runs were performed to assess reproducibility, or inter-run variability. In each run, three replicate standard curves were tested across the 384-well plate to assess repeatability, or intra-run variability. All reactions were performed in triplicates.

#### Data analysis

**Figure 2:**
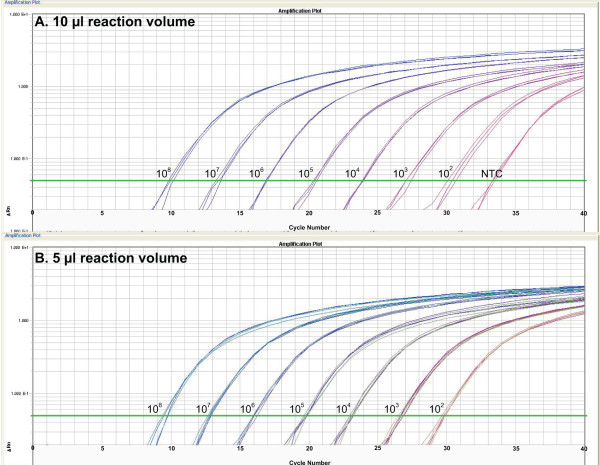
**A-B. Standard curve amplification profiles of the BactQuant assay generated from 10 μl and 5 μl reactions using seven ten-fold dilutions and normalized plasmid standards at 10**^**9**^**copies/μl.** The Ct value of standard curve using 5 μl reaction volumes (Figure2B) shows an approximately 1 Ct left shift from the 10 μl reaction volumes (Figure2A). However, the overall amplification profiles are not significantly different between the different reaction volumes over the assay dynamic range of 10^2^ copies to 10^8^ copies of 16 S rRNA gene per reaction.

**Figure 3:**
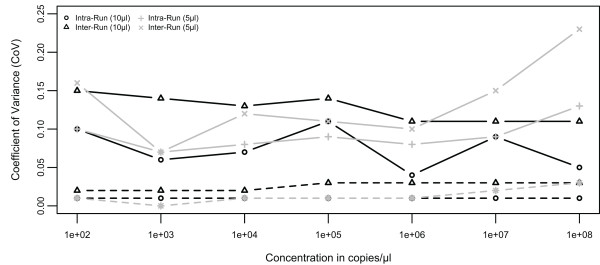
**Inter- and intra-run coefficient of variation (CoV) for 10 μl and 5 μl reactions using seven ten-fold dilutions and normalized plasmid standards at 10**^**9**^**copies/μl calculated using data from multiple runs.** The data is presented for both copy number ( *solid line*) and Ct value ( *dashed line*). As would be expected, the CoV is higher for copy number than for Ct value and is also higher for inter-run than for intra-run. The CoV for copy number for both reaction volumes was consistently below 15% until at 10^7^ copies for 5 μl reactions. The CoV for Ct value was consistently below 5% for both reaction volumes.

Using the data generated, the following assay parameters were calculated: 1) inter-run assay coefficient of variation (CoV) for copy number and Ct value, 2) average intra-run assay CoV for copy number and Ct. value, 3) assay dynamic range, 4) average reaction efficiency, and 5) correlation coefficient (*r*^*2*^-value). The limit of detection was not defined for the pure plasmid standards experiments due to variability in reagent contamination. At each plasmid standard concentration, the Ct standard deviation across all standard curves over three runs was divided by the mean Ct value across all standard curves over three runs to obtain the inter-run assay CoV. The CoV from each standard curve from each run (i.e., nine CoV were used in the calculation for each condition tested) were used to calculate the average and the standard deviation of the intra-run CoV. Linear regression of each standard curve across the full dynamic range was performed to obtain the slope and correlation coefficient values. The slope was used to calculate the reaction efficiency using Efficiency = 10^(−1/slope)^-1. Of note, for each triplicate reaction with Ct standard deviation >0.3, the triplicates were compared and if a clear outlier was present (ΔCt > 0.3 from other two replicates), the outlier well was excluded from analysis. Amplification profiles of the seven conditions tested were annotated and presented in Figure2A-B and Additional file 4: Figure S 4A-E. Results from laboratory quantitative validation using all conditions tested were summarized in Table4. Detailed results of inter- and intra-run coefficient of variation for Ct value and copy number were presented for all conditions tested in Figure3 and Additional file 5: Supplemental file 1A-C using scattered plots generated with the *vegan* package in R [18,19].

**Table 4:** Laboratory quantitative validation results of the BactQuant assay performed using pure plasmid standards and different mixed templates

**Templates used**	**Assay dynamic range**	**Average reaction efficiency (SD)**	***r***^***2***^**–value**
**Plasmid standards–only (10 μl Rxn)**	100–10^8^ copies	102% (2%)	>*0.999*
**Plasmid standards-only (5 μl Rxn)**	100 – 10^8^ copies	95% (1%)	>*0.999*
**Plasmid standards*plus*0.5 ng human gDNA**	100 – 10^8^ copies	99% (4%)	>*0.994*
**Plasmid standards*plus*1 ng human gDNA**	100 – 10^8^ copies	101% (5%)	>*0.994*
**Plasmid standards*plus*5 ng human gDNA**	500 – 10^8^ copies	96% (1%)	>*0.999*
**Plasmid standards*plus*10 ng human gDNA**	1000 – 10^8^ copies	97% (2%)	>*0.999*
**Plasmid standards*plus*0.5 ng** ***C. albicans*gDNA**	100 – 10^8^ copies	97% (1%)	>*0.999*

#### Bacteria-to-human ratio calculations

Calculations were performed using the following copy number and genome size estimates: the average bacterial 16 S rRNA gene copy number per genome was estimated to be 3.94 copies as calculated by rrnDB [20] (accessed at http://ribosome.mmg.msu.edu/rrndb/index.php) and the average human 18 S rRNA gene copy number per genome was estimated to be 400 copies [21]. The diploid human genome was estimated to be 5,758 Mb [22] or the mass equivalent of 5,758 Mb/(0.978×10^3^ Mb/pg) = 5.887 pg per diploid human genome [23].

## Results

### Assay design and initial specificity check

Using our 16 S rRNA gene nucleotide distribution output, we identified a conserved 500 bp region for assay design. Within this region, we selected three highly conserved sub-regions abutting V3-V4 for the design of a TaqMan® quantitative real-time PCR (qPCR) assay (Additional file 6: Supplemental file 2). Degenerate bases were incorporated strategically in the primer sequence to increase the unique 16 S rRNA gene sequence types matching the qPCR assay. No degeneracies were permitted in the TaqMan® probe sequence (Table1). Initial *in silico* specificity analysis using megablast showed that the probe is a perfect match against human and *C. albicans* ribosomal DNA, due to its highly conserved nature, but the primers were specific and screening using human and *C. albicans* genomic DNA did not show non-specific amplification.

### *In silico* analysis of assay coverage using 16 S rRNA gene sequences from 34 bacterial phyla

Numerical and taxonomic *in silico* coverage analyses at the phylum, genus, and species levels were performed using 16 S rRNA gene sequences from the Ribosomal Database Project (RDP) as sequence matching targets. A total of 1,084,903 16 S rRNA gene sequences were downloaded from RDP. Of these, 671,595 sequences were determined to be eligible for sequence match comparison based on sequence availability in the *E. coli* region of the BactQuant assay amplicon. The *in silico* coverage analyses was performed based on perfect match of full-length primer and probe sequences (hereafter referred to as “stringent criterion”) and perfect match with full-length probe sequence and the last 8 nucleotides of primer sequences at the 3′ end (hereafter referred to as “relaxed criterion”).

Using the stringent criterion, *in silico* numerical coverage analysis showed that 31 of the 34 bacterial phyla evaluated were covered by the BactQuant assay. The three uncovered phyla being Candidate Phylum OD1, Candidate Phylum TM7, and Chlorobi (Figure1). Among most of the 31 covered phyla, more than 90% of the genera in each phylum were covered by the BactQuant assay. The covered phyla included many that are common in the human microbiome, such as Tenericutes (13/13; 100%), Firmicutes (334/343; 97.4%), Proteobacteria (791/800; 98.9%), Bacteroidetes (179/189; 94.7%), Actinobacteria (264/284; 93.0%), and Fusobacteria (11/12; 91.7%). Only three covered phyla had lower than 90% genus-level coverage, which were Deferribacteres (7/8; 87.5%), Spirochaetes (9/11; 81.8%), and Chlamydiae (2/9; 22.2%) (Figure1).

On the genus- and species-levels, 1,778 genera (96.2%) and 74,725 species (83.5%) had at least one perfect match using the stringent criterion. This improved to 1,803 genera (97.7%) and 79,759 species (89.1%) when the relaxed criterion was applied (Table2, Additional file 2: Figure S 1). Using the same relaxed criterion, 566,685 or 84% of all eligible sequences were perfect matches with the BactQuant assay (Table2). Detailed taxonomic information on the covered and uncovered OTUs for the BactQuant assay can be found in Additional file 5: Supplemental file 1. Additional file 6: Supplemental file 2.

During our *in silico* validation, a previously published qPCR assay was identified, which was used as a published reference for comparison [15]. The *in silico* comparison showed that the BactQuant assay covers more OTUs irrespective of the criterion applied (Table2, Figure1, Additional file 2: figure S 1). Based on the stringent criterion, the published assay has 10 additional uncovered phyla in comparison to BactQuant; these were: Candidate Phylum OP11, Aquificae, Caldiserica, Thermodesulfoacteria, Thermotogae, Dictyoglomi, Deinococcus-Thermus, Lentisphaerae, Chlamydiae, and Candidate Phylum OP10 (Figure1). Applying the relaxed criterion added two phyla, Aquificae and Lentisphaerae, to those covered by the published assay (Additional file 2: figure S 1). The genus-level coverage of the published assay was also low, with fewer than 50% genus-level coverage in six of its covered phyla. For Cyanobacteria, Planctomycetes, Synergistetes, and Verrucomicrobia, only a single genus was covered by the published assay (Additional file 7: Supplemental file 3). In all, the BactQuant assay covered an additional 288 genera and 16,226 species than the published assay, or the equivalent of 15% more genera, species, and total unique sequences than the published assay (Table2). Detailed taxonomic information on the covered and uncovered OTUs for the published qPCR assay can be found in Additional file 7: Supplemental files 3, Additional file 8: Supplemental files 4.

### Laboratory analysis of assay performance using diverse bacterial genomic DNA

Laboratory evaluation of the BactQuant assay showed 100% sensitivity against 101 species identified as perfect matches from the *in silico* coverage analysis. The laboratory evaluation was performed using genomic DNA from 106 unique species encompassing eight phyla: Actinobacteria (n = 15), Bacteroidetes (n = 2), Deinococcus-Thermus (n = 1), Firmicutes (n = 18), Fusobacteria (n = 1), Proteobacteria (n = 66), Chlamydiae (n = 2), and Spirochaetes (n = 2). Overall, evaluation using genomic DNA from the 101 *in silico* perfect match species demonstrated *r*^*2*^-value of >0.99 and amplification efficiencies of 81 to 120% (Table3).

Laboratory evaluation against the five *in silico* uncovered species showed variable assay amplification profiles and efficiencies. Of these five species, *Chlamydia trachomatis*, *Chlamydophila pneumoniae*, and *Cellvibrio gilvus* were identified as uncovered irrespective of *in silico* analysis criterion. However, while *C. trachomatis* and *C. pneumoniae* showed strongly inhibited amplification profile, *C. gilvus* amplified successfully with a *r*^*2*^*-*value of >0.999 and an amplification efficiency of 92% (Additional file 3: Figure S 3A-B & 3E). Two other species, *Borrelia burgdorferi* and *Escherichia vulneris*, which were uncovered only when using the stringent criterion, also showed successful amplification with a *r*^*2*^*-*value of >0.999 and 90% and 93% reaction efficiency, respectively (Additional file 3: Figure S 3C-D). Comparison of the assay and bacterial sequences showed that *C. trachomatis* and *C. pneumoniae* shared a single mismatch in the center of the probe sequence, whereas *C. gilvus* had a mismatch on the 3′ end of the probe. The mismatch in *B. burgdorferi* and *E. vulneris* was a single base difference in 5′ end of the reverse and the forward primer, respectively (Additional file 3: Figure S 3A-E). These findings strongly suggest the location of the sequence mismatch is an important determinant of amplification outcome. Furthermore, it supports that the BactQuant assay’s coverage in laboratory application is likely greater than determined by the *in silico* analyses.

### Laboratory quantitative assay validation using pure plasmid standards and mixed templates

To fully characterize the assay quantitative profile, the BactQuant assay was tested using different reaction volumes and against both pure and mixed templates containing bacterial and human rRNA gene targets. Laboratory evaluation using pure plasmid standards in 10 μl and 5 μl reaction volumes showed excellent amplification profiles, with an assay dynamic range of 10^2^–10^8^ 16 S rRNA gene copies per reaction (Figure2A–B). For the 10 μl reactions, the inter- and intra-run coefficients of variance (CoV) ranged from 1.58–2.94% and 0.64–1.25% for Ct values and from 10.60–15.36% and 4.02–10.51% for copy number, respectively (Figure3). The inter- and intra-run CoV was comparable for the different reaction volumes, except for the higher CoV in 5 μl reactions containing more than 10^7^ plasmid copies (Figure3). This suggests that the 5 μl reaction volumes may be better suited for samples with low amounts of bacterial DNA. Establishment of the limit of detection (LOD) for the BactQuant assay using pure plasmid standards was not attempted because it was affected by the level of contaminants in reagents, as previously reported [15,24-28].

Further laboratory evaluations using mixed templates showed that the ratio of bacteria-to-human DNA ratio determined the assay dynamic range of the BactQuant assay (Table4, Additional file 4: Figure S 4A-E, Additional file 5: Additional file 9: Table S 1A–C). Experiments using seven tenfold dilutions of plasmid standards with 0.5 ng and 1 ng human gDNA showed that the assay dynamic range was unchanged from pure plasmid standard. However, experiments using 5 ng and 10 ng of human gDNA showed narrower assay dynamic ranges of 500 - 10^8^ and 1000 - 10^8^ 16 S rRNA gene copies per reaction, respectively. Based on this result, the LODs for 10 μl reactions using templates containing 5 ng and 10 ng of human gDNA were estimated to be a bacteria-to-human ribosomal gene copy ratio of 500:339732 and 1000:679464, respectively. This could be further simplified to a bacteria-to-human ribosomal gene copy ratio of 1:679. From a genomic equivalent perspective, the LOD of the BactQuant assay was approximately at a bacteria-to-human ratio of 127:849.

## Discussion

We designed and evaluated a new expanded-coverage bacterial quantitative real-time PCR assay targeting the 16 S rRNA gene. To accomplish this, we curated a set of high-quality 16 S rRNA gene sequences for assay design and evaluated the coverage of our primers and as a union (rather than as separate entities). In addition, we improved the quantitative capacity of our assay using a cloned plasmid standard. Our computational and laboratory analyses showed that BactQuant had superior *in silico* taxonomic coverage while retaining favorable in vitro performance. As would be expected, the diverse gene sequences targeted by BactQuant have resulted in variable reaction efficiencies. Nevertheless, laboratory evaluation showed 100% sensitivity against perfect match species from the *in silico* analysis.

To allow researchers to determine whether BactQuant covers key organisms in their target community, we provided additional detailed OTU coverage information in the Supplemental Files. We have applied the logic that an OTU was covered if it contained at least one perfect match sequence in the *in silico* analysis. 16 S rRNA gene sequences with ambiguous or degenerate bases at the primer and probe sites were considered non-perfect matches, thus making our coverage estimates more conservative. Lastly, although we prohibited the use of a degenerate probe to maximize our assay’s quantitative ability, this approach may permit detection of specific taxa such as *Chlamydia* spp *.* and *Chlamydophila* spp.

For most studies, the desired measurement of bacterial load is the number of cells rather than 16 S rRNA gene copy number; however, the 16 S rRNA gene copy number varies among bacterial species and even among strains [29,30]. The range of copy number is estimated at one to 14, with most non-spore forming species having fewer than 10 copies per genome [20]. We use the average 16 S rRNA gene copy number per genome from rrnDB in our genomic equivalent estimation, but alternative approaches are possible. This, combined with logarithmic growth of bacteria, suggest that using estimated average copy number could be sufficient.

The *in silico* analysis was an important component of our validation of BactQuant against diverse bacterial sequence types, even though sequence matching is not a perfect predictor of laboratory performance [31]. Many factors are known to affect reaction efficiency, such as oligonucleotide thermodynamics, the type of PCR master mix used, and the template DNA extraction method. Concentration of background nontarget genomic DNA is another factor that can affect the quantitative parameters rRNA gene-based assays [32]. The interference of background human DNA with BactQuant dynamic range reported in this paper was most likely due to cross-reactivity of human DNA with the probe, which targets a region conserved even among eukaryotic organisms, including in the human 18 S rRNA gene. This may be overcome by using an intercalating reporter dye in place of a fluorescent probe as a qPCR reporter mechanism; however, the loss of tertiary-level of specificity is a potential concern in direct application of an intercalating dye assay to specimens containing high amounts of nontarget DNA.

Exogenous bacterial DNA, particularly from biologically synthesized reagents such as Taq DNA polymerase are a known limitation for analyzing samples with low bacterial load [28,33]. Recently, this issue has received renewed attention due to increased usage of next-generation sequencing and the frequent data contamination from exogenous bacterial DNA. Several methods have been evaluated for removing bacterial contaminants from Taq DNA polymerase, including UV irradiation [34,35], DNAse I treatment, and ultrafiltration [36]. The level of *E. coli* contamination in Taq DNA polymerase has been estimated at 10^2^ to 10^5^ genome equivalents of bacterial DNA per unit of enzyme [28]. This is consistent with the lowest amount of contamination we have observed in our experiments, which were 5 and 10 copies of 16 S rRNA gene in 5 μl and 10 μl reactions, respectively. The ubiquity of bacterial DNA also makes the determination of assay specificity challenging.

Our use of qPCR-quantified plasmid standards addressed a major limitation in the preparation of qPCR quantification standards. The conventional approach of quantifying bacterial genomic DNA or plasmid standards necessitates converting mass (i.e., nanograms per μl) to copy number (i.e., 10^8^ copies per μl) and can introduce substantial error. Thus far, we have also successfully applied BactQuant to a diverse range of clinical specimens, including swab eluents, surgical specimens, and respiratory specimens, but we did not present these findings in this paper. To fully understand the likelihood of false negative results due to interference from human DNA or BactQuant’s limit of detection will require additional evaluations.

## Conclusion

In summary, we have developed and evaluated a new broad-coverage qPCR assay—BactQuant—for bacterial detection and quantification that showed concurrently improved assay coverage and favorable quantitative parameters. Laboratory tests showed that in vitro performance was even better than predicted in the *in silico* analysis; however, our approach of evaluating assay coverage by considering the primer and probe sequences as a single unit is appropriate and necessary. In addition, when employing a copy number estimation method, such as qPCR, the quantification of standards is critical for accurate template quantification. Thus, our approach of quantifying plasmid standards using the intrinsic property of real-time PCR is another important step for any absolute quantification experiments using qPCR.

## Competing interests

The authors have declared that no competing interests exist.

## Authors’ contributions

CML contributed to the overall study design, the acquisition, analysis, and interpretation of data, and drafting the manuscript, MA contributed to the bioinformatics portion of the study design and its implementation, SK participated in bioinformatics analysis and assay design, PRH and YTH both contributed to the acquisition and interpretation of laboratory data, PK conceived of the study and contributed to the overall study design, LBP contributed to the overall study design and helped to draft the manuscript. All authors read and approved the final manuscript.

## Supplementary Material

Additional file 1 **Figure S1.** Figure S1 containing the *in silico* coverage analysis using the relaxed criteria. Click here for file

Additional file 2 **Figure S2A-E.** Standard curve amplification plots using mixed templates. Click here for file

Additional file 3 **Figure S3A-E.** Amplification plots of the non-perfect match targets, including *C. trachomatis, C. pneumoniae, C. gilvus, B. burgdorferi,* and *E. vulneris*. Click here for file

Additional file 4 **Figure S4A-E.** Coefficient of variance (CoV) distribution across assay dynamic range for mixed templates. Click here for file

Additional file 5 **Supplemental File 1.** Detailed results for BactQuant using the stringent criteria. Click here for file

Additional file 6 **Supplemental File 2.** Detailed results for BactQuant using the relaxed criteria. Click here for file

Additional file 7 **Supplemental File 3.** Detailed results for published assay using the stringent criteria. Click here for file

Additional file 8 **Supplemental File 4.** Detailed results from published assay using the relaxed criteria. Click here for file

Additional file 9 **Table S1.** Base distribution output used in primer and probe design, with the bolded base signifying the selected base(s) and incorporation of more than one allele at a given nucleotide position was accomplished using degenerate bases. The alignment position information in the base distribution file contains many gaps as a result from the sequence alignment and differs from the *E. coli* region information from Table 1. Click here for file
