# Supplementary material for: BactQuant: An enhanced broad-coverage bacterial quantitative real-time PCR assay
Source: BMC Microbiol. 2012 Apr 17;12:56. doi: 10.1186/1471-2180-12-56 (PMC3464140; doi:10.1186/1471-2180-12-56)
Supplement: Additional file 1 — Figure S1. Figure S1 containing the in silico coverage analysis using the relaxed criteria. [file 1471-2180-12-56-S1.doc]

**SUPPLEMENTARY FIGURE LEGENDS**

**Fig. S1.** **Results from *in silico* coverage analysis of the BactQuant assay using the relaxed criterion against 1,849 genera and 34 phyla showing broad coverage improved from stringent criterion.** The number of genera covered in each phylum analyzed (*left*) and the list of all uncovered genera (*right*) are shown. On the circular 16S rRNA gene-based maximum parsimony phylogeny (*left*), each covered (*in black*) and the uncovered (*in red*) phylum is annotated with the genus-level numerical coverage in parenthesis below the phylum name. The genus-level numerical coverage presented consists of a numerator (i.e., the number of covered genus for the phylum), a denominator (i.e., the total number of genera eligible for sequence matching for the phylum), and a percentage calculated using the numerator and denominator values. Coverage comparison with the published qPCR assay is presented for each phylum as notations of a single asterisk (*) for phylum not covered by the published qPCR assay and as a double asterisk (**) for phylum with <50% of its genera covered by the published qPCR assay. The phylum and genus taxonomic identification of all uncovered genera are also presented (*right*).

**Fig. S2A-E.** **Standard curve amplification profiles of the BactQuant assay generated from 10 µl using seven ten-fold dilutions and mixed templates consisting of human or fungal genomic DNA mixed with normalized plasmid standards.** The human or fungal genomic DNA added per reaction was 0.5 ng human (Fig. S2A), 1 ng human (Fig. S2B), 5 ng human (Fig. S2C), 10 ng human (Fig. S2D), and 0.5 ng *C. albicans* (Fig. S2E). The amplification profiles of reactions from plasmid standards of higher amounts at >10^4^ copies per reaction were generally unchanged from those using pure plasmid standards. However, at bacteria to human rRNA gene ratio of 100:33973 (i.e., 100 copies of plasmids mixed with 0.5 ng of human gDNA) and 100:67946 (i.e., 100 copies of plasmids mixed with 1 ng of human gDNA) the amplification profile begins to have a lower plateau. By bacteria to human rRNA gene ratio of 100:339732 (i.e., 100 copies of plasmids mixed with 5 ng of human gDNA) and 100:679464 (i.e., 100 copies of plasmids mixed with 10 ng of human gDNA), the amplification profile was strongly inhibited. This experiment showed that it is the ratio of bacteria and non-target eukaryotic DNA that affects the lower limit of detection and assay dynamic range. Thus, samples with higher bacterial load are less likely to become inhibited by the presence of non-target eukaryotic DNA.

**Fig. S3A-E. Amplification profiles and sequence comparison for five uncovered bacterial species tested demonstrating the impact of mismatch location on amplification profile and efficiency.** *Chlamydia trachomatis* (Fig. S3A) and *Chlamydophila pneumonia* (Fig. S3B) each had a mismatch in the center of the probe sequence and showed significant inhibited amplification profiles. In contrast, *Cellvibrio gilvus* (Fig. S3C), which had a single mismatch on the 3’ end of the probe did not exhibit the same level of inhibition and has an amplification efficiency of 92%. *Borrelia burgdorferi* (Fig. S3D) and *Escherichia vulneris* (Fig. S3E), which had a single mismatch in the 5’ end of the reverse and the forward primer sequence, respectively, did not show significant inhibition. *B. burgdorferi* and *E. vulneris* were both identified as matches in the relaxed *in silico* analysis.

**SUPPLEMENTAL TABLE**

**Table S1.** Base distribution output used in primer and probe design, with the bolded base signifying the selected base(s) and incorporation of more than one allele at a given nucleotide position was accomplished using degenerate bases. The alignment position information in the base distribution file contains many gaps as a result from the sequence alignment and differs from the *E. coli* region information from Table 1.

| Alignment Nucleotide Position | A | T | C | G | Ambiguous Base | No. of Gaps |
| --- | --- | --- | --- | --- | --- | --- |
| **Forward Primer** | | | | | | |
| Position 1872: | 10 | 84 | **4802** | 6 | 1 | 35 |
| Position 1873: | 7 | 38 | **4803** | 10 | 3 | 77 |
| Position 1874: | 0 | 3 | 87 | 1 | 0 | 4847 |
| Position 1875: | 6 | **4906** | 20 | 0 | 0 | 6 |
| Position 1876: | **4837** | 22 | 15 | 63 | 0 | 1 |
| Position 1877: | 10 | 31 | **4890** | 7 | 0 | 0 |
| Position 1878: | 0 | 0 | 3 | 1 | 0 | 4934 |
| Position 1879: | 15 | 11 | 27 | **4882** | 2 | 1 |
| Position 1880: | 0 | 0 | 0 | 0 | 0 | 4938 |
| Position 1881: | 0 | 1 | 0 | 0 | 0 | 4937 |
| Position 1882: | 10 | 5 | 5 | **4917** | 0 | 1 |
| Position 1883: | 3 | 0 | 0 | 0 | 0 | 4935 |
| Position 1884: | 80 | 15 | 8 | **4833** | 0 | 2 |
| Position 1885: | 1 | 1 | 1 | 5 | 0 | 4930 |
| Position 1886: | **4408** | **250** | 6 | **271** | 1 | 2 |
| Position 1887: | 2 | 0 | 0 | 0 | 0 | 4936 |
| Position 1888: | 18 | 38 | 149 | **4731** | 2 | 0 |
| Position 1889: | 2 | 2 | 1 | 3 | 0 | 4930 |
| Position 1890: | 13 | 9 | 15 | **4899** | 1 | 1 |
| Position 1891: | 5 | 14 | **4903** | 16 | 0 | 0 |
| Position 1892: | 1 | 0 | 3 | 0 | 0 | 4934 |
| Position 1893: | **4618** | **283** | 19 | 12 | 0 | 6 |
| Position 1894: | 3 | 1 | 1 | 3 | 0 | 4930 |
| Position 1895: | 26 | 9 | 53 | **4846** | 3 | 1 |
| Position 1896: | 5 | 9 | **4907** | 9 | 2 | 6 |
| Position 1897: | 1 | 2 | 2 | 0 | 0 | 4933 |
| Position 1898: | **4882** | 17 | 23 | 10 | 1 | 5 |
| **Probe (targets the reverse strand because of its proximity to the F primer; no degeneracy allowed)** | | | | | | |
| Position 2233: | 6 | 6 | **4913** | 4 | 1 | 8 |
| Position 2234: | **4887** | 10 | 32 | 5 | 0 | 4 |
| Position 2235: | 10 | 1 | 0 | 1 | 0 | 4926 |
| Position 2236: | 5 | 1 | 0 | 0 | 0 | 4932 |
| Position 2237: | 30 | 10 | 33 | **4847** | 3 | 15 |
| Position 2238: | 0 | 2 | 0 | 3 | 0 | 4933 |
| Position 2239: | 48 | 7 | **4875** | 3 | 1 | 4 |
| Position 2240: | 0 | 0 | 1 | 0 | 0 | 4937 |
| Position 2241: | 0 | 0 | 0 | 0 | 0 | 4938 |
| Position 2242: | 1 | 0 | 5 | 0 | 0 | 4932 |
| Position 2243: | **4717** | 8 | 195 | 15 | 0 | 3 |
| Position 2244: | 0 | 0 | 0 | 2 | 0 | 4936 |
| Position 2245: | 2 | 2 | 10 | 19 | 0 | 4905 |
| Position 2246: | 27 | 15 | 39 | **4853** | 1 | 3 |
| Position 2247: | 4 | 40 | **4857** | 26 | 0 | 11 |
| Position 2248: | 65 | 35 | **4809** | 23 | 0 | 6 |
| Position 2249: | 5 | 42 | 45 | **4835** | 0 | 11 |
| Position 2250: | 0 | 0 | 2 | 33 | 0 | 4903 |
| Position 2251: | 0 | 0 | 2 | 1 | 0 | 4935 |
| Position 2252: | 0 | 1 | 5 | 2 | 0 | 4930 |
| Position 2253: | 2 | 11 | **4901** | 18 | 0 | 6 |
| Position 2254: | 3 | 0 | 4 | 10 | 0 | 4921 |
| Position 2255: | 13 | 6 | 13 | **4889** | 0 | 17 |
| Position 2256: | 6 | 6 | 6 | **4909** | 0 | 11 |
| Position 2257: | 0 | 0 | 0 | 1 | 0 | 4937 |
| Position 2258: | 1 | 10 | 1 | 11 | 0 | 4915 |
| Position 2259: | 10 | **4881** | 15 | 23 | 0 | 9 |
| Position 2260: | **4844** | 20 | 21 | 50 | 1 | 2 |
| **Reverse Primer (shown as gene target sequence)** | | | | | | |
| Position 4050: | 63 | 1 | 9 | **4851** | 1 | 13 |
| Position 4051: | 13 | 0 | 1 | 7 | 0 | 4917 |
| Position 4052: | **4875** | 20 | 26 | 10 | 0 | 7 |
| Position 4053: | 15 | **4902** | 9 | 9 | 0 | 3 |
| Position 4054: | 19 | **4899** | 7 | 9 | 0 | 4 |
| Position 4055: | **4899** | 15 | 1 | 16 | 0 | 7 |
| Position 4056: | 17 | 0 | 0 | 1 | 0 | 4920 |
| Position 4057: | 30 | 6 | 3 | **4896** | 1 | 2 |
| Position 4058: | 3 | 0 | 2 | 1 | 0 | 4932 |
| Position 4059: | **4914** | 12 | 0 | 10 | 0 | 2 |
| Position 4060: | 67 | **4747** | 10 | 113 | 0 | 1 |
| Position 4061: | **4863** | 19 | 22 | 14 | 2 | 18 |
| Position 4062: | 25 | 1 | 26 | 0 | 0 | 4886 |
| Position 4063: | 4 | 9 | **4893** | 2 | 0 | 30 |
| Position 4064: | 0 | 0 | 0 | 0 | 0 | 4938 |
| Position 4065: | 0 | 0 | 0 | 0 | 0 | 4938 |
| Position 4066: | 0 | 0 | 0 | 0 | 0 | 4938 |
| Position 4067: | 0 | 0 | 0 | 0 | 0 | 4938 |
| Position 4068: | 0 | 2 | 10 | 0 | 0 | 4926 |
| Position 4069: | 4 | 28 | **4898** | 6 | 0 | 2 |
| Position 4070: | 7 | 72 | **4839** | 13 | 0 | 7 |
| Position 4071: | 0 | 5 | 25 | 2 | 0 | 4906 |
| Position 4072: | 9 | **3729** | **866** | **328** | 0 | 6 |
| Position 4073: | 0 | 3 | 4 | 5 | 0 | 4926 |
| Position 4074: | **181** | **142** | 64 | **4544** | 1 | 6 |
| Position 4075: | 0 | 1 | 2 | 12 | 0 | 4923 |
| Position 4076: | 9 | 21 | 6 | **4872** | 0 | 30 |
| Position 4077: | 3 | **4916** | 5 | 9 | 0 | 5 |
| Position 4078: | **4905** | 10 | 2 | 18 | 1 | 2 |
| Position 4079: | 6 | 4 | 0 | 1 | 0 | 4927 |
| Position 4080: | 28 | 25 | 6 | **4877** | 0 | 2 |
| Position 4081: | 0 | 8 | 0 | 3 | 0 | 4927 |
| Position 4082: | 5 | **4912** | 12 | 4 | 0 | 5 |
| Position 4083: | 0 | 0 | 0 | 0 | 0 | 4938 |
| Position 4084: | 0 | 0 | 0 | 0 | 0 | 4938 |
| Position 4085: | 0 | 1 | 1 | 0 | 0 | 4936 |
| Position 4086: | 2 | 0 | 5 | 0 | 0 | 4931 |
| Position 4087: | 10 | 27 | **4879** | 9 | 0 | 13 |
| Position 4088: | 1 | 0 | 24 | 0 | 0 | 4913 |
| Position 4089: | 5 | 73 | **4845** | 8 | 1 | 6 |
