# Supplementary figures and images for: BactQuant: An enhanced broad-coverage bacterial quantitative real-time PCR assay
Source: BMC Microbiol. 2012 Apr 17;12:56. doi: 10.1186/1471-2180-12-56 (PMC3464140; doi:10.1186/1471-2180-12-56)

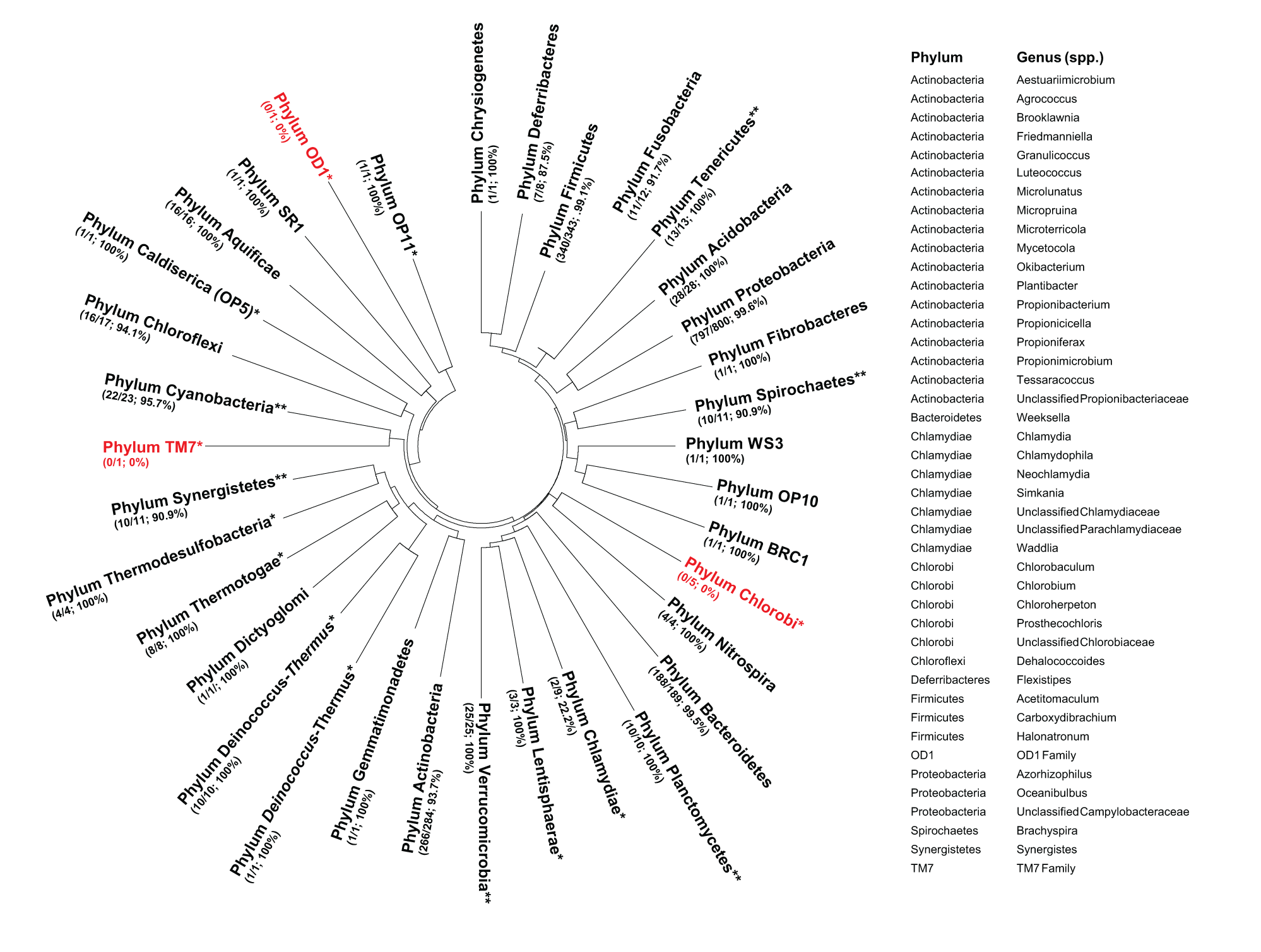

Supplement: Additional file 2 — Figure S2A-E. Standard curve amplification plots using mixed templates. [file 1471-2180-12-56-S2.tiff]

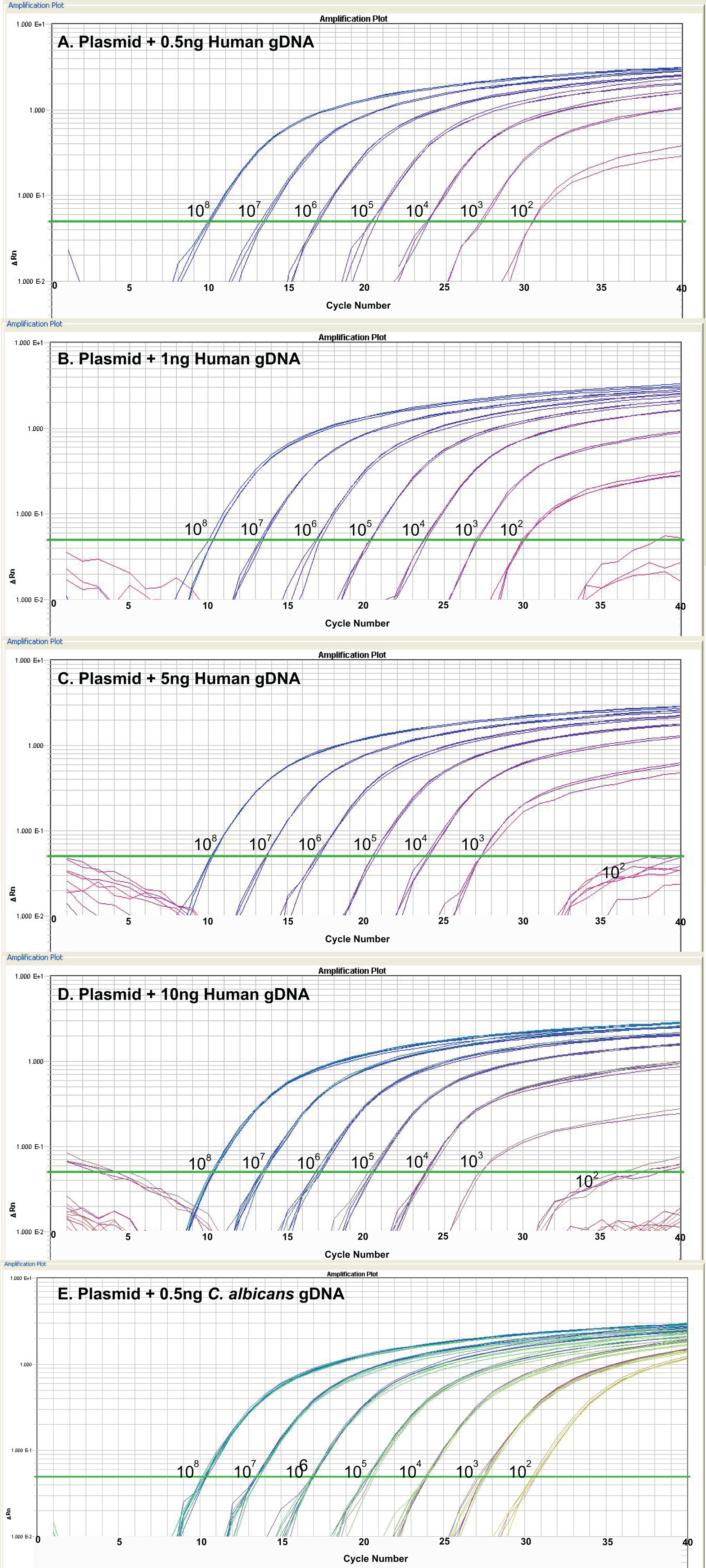

Supplement: Additional file 3 — Figure S3A-E. Amplification plots of the non-perfect match targets, including C. trachomatis, C. pneumoniae, C. gilvus, B. burgdorferi, and E. vulneris. [file 1471-2180-12-56-S3.tiff]

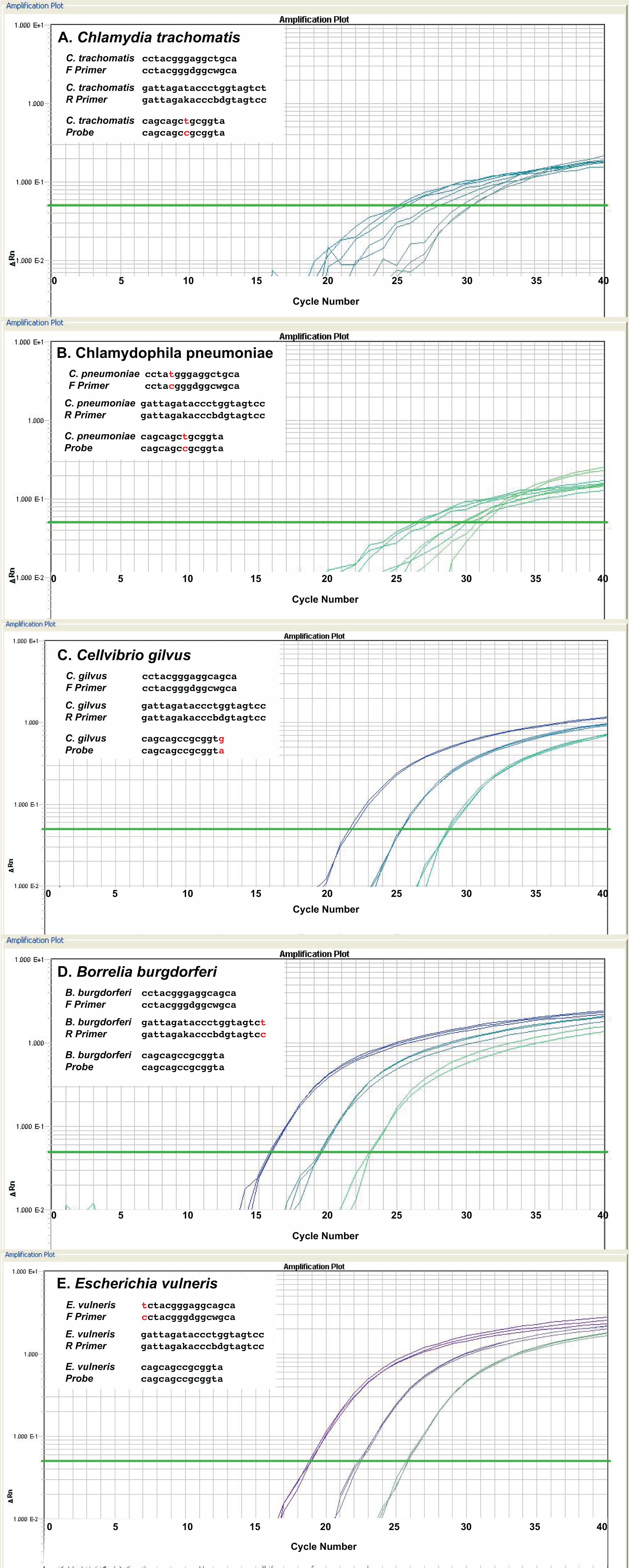

Supplement: Additional file 4 — Figure S4A-E. Coefficient of variance (CoV) distribution across assay dynamic range for mixed templates. [file 1471-2180-12-56-S4.tiff]

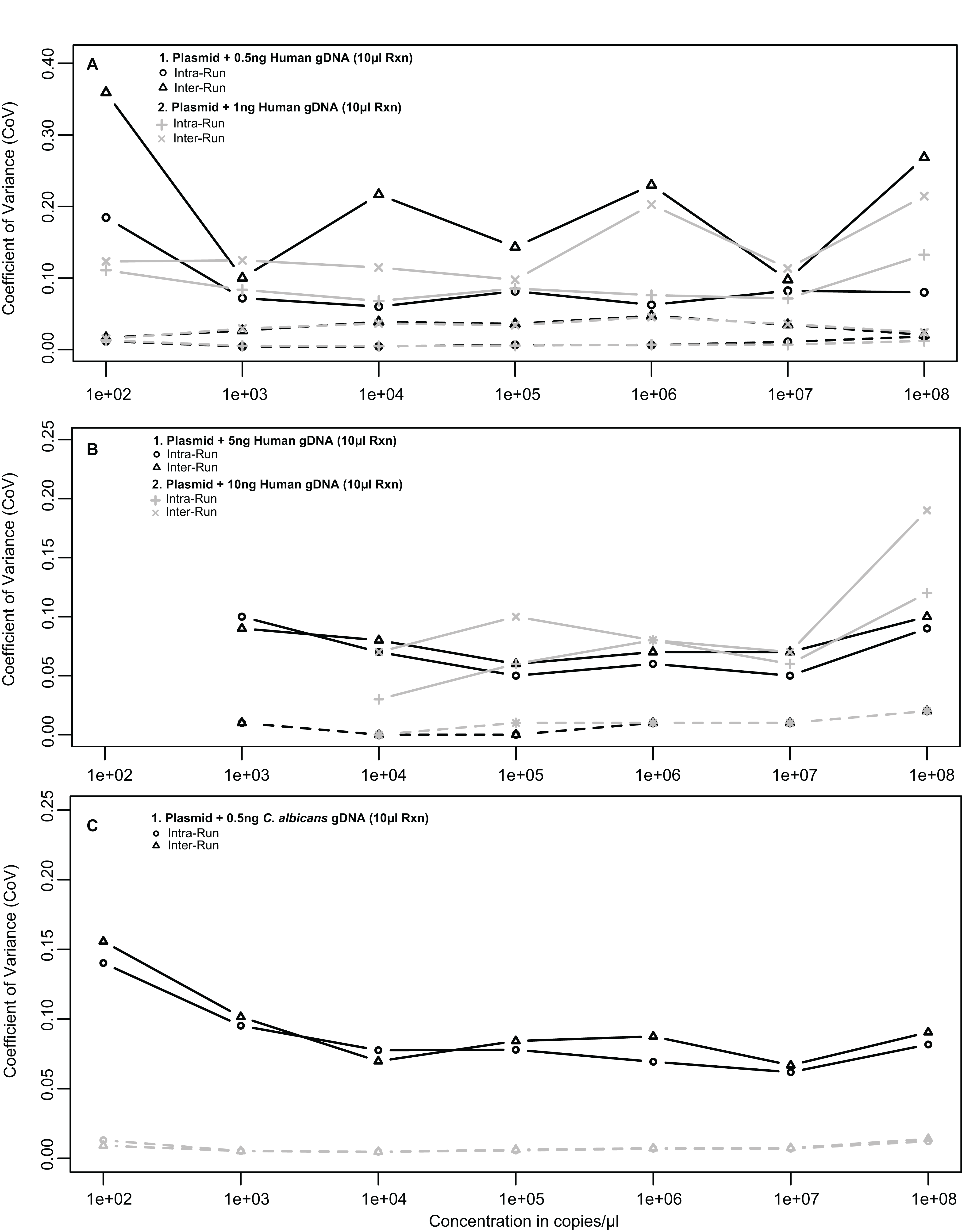

Supplement: Additional file 5 — Supplemental File 1. Detailed results for BactQuant using the stringent criteria. [file 1471-2180-12-56-S5.tiff]
